# Supplementary material for: Are carbon emissions trading and green financial instruments synergistic? -Comprehensive quantitative research based on content analysis
Source: PLoS One. 2024 Mar 7;19(3):e0298601. doi: 10.1371/journal.pone.0298601 (PMC10919601; doi:10.1371/journal.pone.0298601)
Supplement: S1 Data — (ZIP) [file pone.0298601.s001.zip › Data/地级市Z_F工作报告.zip!/╣≤╓▌/┴∙┼╠╦«╩╨/└┤╫╘╣1⁄2╓┌║┼í╛┬φ┐╦╩2╛▌═°í┐_2013.docx]

各位代表：

　　现在，我代表市人民政府向大会报告工作，请予审议，并请各位政协委员和列席的同志提出意见。

　　2012年政府工作回顾

　　过去一年，在省委、省政府和市委的坚强领导下，在市人大、市政协的监督、支持和帮助下，我们坚持以科学发展观为统领，以贯彻落实国发2号文件为总抓手，认真贯彻落实省第十一次党代会、市第六次党代会精神，坚持“两加一推”主基调，大力实施“三化同步”主战略，着力保增长、调结构、增活力、惠民生、促和谐，努力克服经济下行压力加大等不利因素的影响，全市呈现出“发展提速、投资增加、民生改善、后劲增强、社会和谐、人心思进”的良好局面，较好地完成了年初确定的目标任务。

　　全市生产总值完成738.65亿元，增长16%，增速高于全国、高于西部、高于全省。财政总收入完成156.35亿元，增长21.71%；公共财政预算收入完成103.49亿元，增长46.11%。社会消费品零售总额完成182.82亿元，增长16%。城镇居民人均可支配收入达到18764元，增长14.6%；农民人均纯收入达到5182元，增长16.8%。金融机构人民币存、贷款余额分别为676.21亿元、508.38亿元，分别增长17.19%、21.92%。全市综合排位实现前移进位，从2011年末的全省第6名上升至第4名。

　　一年来，我们主要抓了以下八个方面的工作。

　　（一）强力推进项目建设，固定资产投资大幅增加。着力抓好国发2号文件的项目化、实物化落实，积极“上争”，全年争取中央预算内资金15.54亿元，是近年来争取中央资金最多的一年。通过建立“三化”现场观摩会、项目集中开工和固定资产投资“旬调度、月督查、季考核”等项目推进机制，项目建设掀起新高潮。全社会固定资产投资突破千亿元大关，达到1088.9亿元，增长97.8%，其中50万元以上固定资产投资完成766.39亿元，增长74.2%。81个省级重点项目完成投资147.7亿元，329个市级重点项目完成投资428亿元。分季度集中新开工项目累计940个、总投资1193.3亿元，完成投资334亿元。加大项目前期工作力度，市级投入项目前期工作经费8400万元，增长320%，项目库储备项目达3518个。多渠道筹集项目建设资金，大力推行企业债券、信托融资、中期票据、融资租赁等融资方式，实现直接融资49.24亿元。尽力保障项目建设用地，全年供应土地4346.77公顷，增长375.9%。

　　（二）努力克服经济下行影响，工业经济溯逆而进。通过采取调整产品结构、价跌量补、高载能行业配套电价补贴等应对措施，下大力抓扶持、促生产、拓市场，工业经济实现平稳较快发展。全市规模以上工业增加值364.42亿元，增长21.6%。传统支柱产业进一步做大做强，主要工业产品产量实现稳步提升，原煤产量7156万吨、钢产量501.48万吨、发电量375.49亿千瓦时、水泥产量523.24万吨。着力打造煤电钢、煤电铝、煤电化、煤电材“四个一体化”品牌，六枝路喜、黔桂天能等一批重点循环经济项目加快推进。西南天地煤炭装备制造等一批新兴产业不断壮大。产业园区建设取得新进展，增加值占工业增加值的42.5%，基础设施建设投入完成60.8亿元，建成标准厂房120.4万平方米，在建企业165家，投产企业149家。更加注重节能减排，万元GDP能耗降低3.43%，完成了主要污染物总量减排目标任务。

　　（三）加大城镇规划建设力度，城镇化进程不断加快。紧紧围绕打造现代特色都市目标，以承办第八届全省旅游产业发展大会为契机，加快城镇建设步伐，城乡面貌改观较大，城镇化率提高到41%。启动第四轮城市总体规划修编，完成30个乡镇、505个村的村庄整治规划编制工作。市中心城区基础设施和公共服务设施建设力度进一步加大，以城市综合体引领城市发展，启动了凤凰山、六盘水体育中心、明湖等一批各具特色的城市综合体项目，市中心城区“6个城市广场”、“6个城市湿地公园”建设步伐加快，市中心城区集中供热工程启动实施。红桥新区、双水城区、六枝城区、盘县城区的城市建设取得较大进展。以郎岱、柏果、玉舍等10个省级、市级特色示范小城镇建设为重点，小城镇建设步伐加快。深入开展“整脏治乱”、“五城联创”活动和“五项城市管理工程”，城市管理水平进一步提升。

　　（四）坚持以农民增收为核心，农业农村经济加快发展。认真落实强农惠农政策，争取省级以上农业资金16.04亿元，市、县财政共投入农业专项资金3.23亿元，农业农村投资大幅增加。全年粮食获得丰收，总产量达75.29万吨，增长33.4%。特色产业发展加快，“九大产业”均按计划或超额完成，结构调整取得实质性进展，粮经比达到63:37。六枝郎岱、盘县滑石、水城米箩、钟山大河等一批农业产业园区加快建设。农业产业化经营步伐加快，新增省级龙头企业14家、市级龙头企业19家，农民专业合作经济组织402个，4家中药制药企业通过国家GMP认证。首次举办农业产业重点项目推介暨投资洽谈会，签约资金44.9亿元，22个签约项目开工建设。全年完成土地流转10.45万亩。深入推进“四在农家”创建工作，完成6万户新民居建设改造任务，农村面貌发生显著变化。

　　（五）加强基础设施建设，发展条件进一步改善。六盘水月照机场飞行区土石方主体工程基本完成；长昆铁路客运专线建设进展顺利，六沾铁路复线建成通车，六盘水火车站站房改造主体工程基本建成；水盘高速公路进入收尾阶段，六镇高速、六六高速、杭瑞高速境内段建设有序推进，机场高速、市中心城区内环快线开工建设，启动了盘兴高速、六威高速、毕兴高速前期工作；建成乡村公路756.7公里。鱼洞坝、白河沟水库基本建成，双桥、旧院、卡河、观音岩、懒龙河等中型水库建设顺利；市中心城区应急水源花鱼洞供水工程开工建设；实施病险水库除险加固5座，完成烟水配套工程1.5万亩，新增有效灌溉面积4.86万亩，解决了22.79万农村人口饮水安全问题。完成信息基础设施和电网建设投资12.75亿元。深入实施天然林保护、石漠化治理等重点生态工程，完成营造林36.33万亩。

　　（六）继续深化改革开放，发展动力活力明显增强。国有企业改革改制工作有序、规范推进。投融资体制改革取得新突破，成立了市政府金融办，新组建的8家市级融资平台公司、3家村镇银行运行良好，全市首支16亿元企业债券发行成功。改革试点示范工作取得新进展，钟山区和水城县滥坝镇被列入全国第三批改革发展试点县、试点城镇，水城县被确定为全省统筹城乡和新型工业化试点县。市、县两级政府政务服务大厅建成投运，减少126项行政许可事项、106项非行政许可事项、184项行政服务事项，行政效能进一步提高。公共财政体系初步建立。文化、卫生、教育事业单位实现全员聘用制。民营经济不断壮大，完成增加值338.93亿元，占全市生产总值的比重达45.89%。深化区域经济交流与合作，成功举办“第三届川滇黔十市州合作与发展峰会”暨“第九届中国凉都·六盘水消夏文化节”。招商引资力度加大，成功引进了中建四局、川威集团、富力集团、葛洲坝集团等一批战略合作者，全年招商引资实际到位资金650.07亿元，增长174.3%。

　　（七）加快发展社会事业，公共服务水平不断提高。教育“四项突破工程”全面实施，新建、改扩建乡镇幼儿园25所，启动建设县区中学5所、小学6所，建成农村寄宿制学校3万平方米、教师周转房3598套，实施农村中小学学生营养餐工程、惠及21.3万名学生。贵州科学院六盘水分院正式授牌，国家煤炭清洁转化产品质量监督检验中心基本建成。市人民医院成功创建三级甲等医院，水矿医院等3家创建三级甲等医院通过省级评审；乡镇卫生院和村卫生室规范化建设稳步推进；各类传染病得到有效控制。人口计生工作实现“三降一升”，摆脱了全省挂末位次。大力实施文化、体育、广电惠民工程，六盘水大剧院、市博物馆、市图书馆、六盘水美术馆等项目开工建设；建成全民健身路径25条、村级农民体育健身工程35个、农家书屋125个、农村数字书屋20个；完成24789套农村广播电视设备安装，新增58个行政村有线电视联网延伸覆盖。双拥工作成效显著。妇女、儿童、老龄、残疾人等事业健康发展，科技、民族、宗教、外事、侨务、对台、档案、气象、人防、共青团、工会、红十字等工作取得新成绩。

　　（八）高度重视民生问题，发展成果更多惠及人民群众。扎实办好“十二件民生实事”，完成投资41.9亿元。全面实施积极的就业政策，省级创业型城市创建工作通过评审；认真落实“3个15万元”政策，新增微型企业1600户，带动就业10751人；新增城镇就业86374人，城镇登记失业率为4.14%。基本医疗、基本养老、失业、工伤、生育等保险制度体系进一步完善；城镇低保月人均保障标准提高40元，农村低保年平均保障标准提高157元，保障水平位居全省前列。新建48个居家养老服务站（点）和9个农村养老服务中心，市老年公寓投入使用。加强保障性住房建设，完成14284套保障性安居工程和33090户农村危房改造任务；启动实施150万平方米城市棚户区改造；利用公积金贷款支持保障性住房7.93亿元，被国家列为公积金支持保障性住房建设试点城市。全力打造“扶贫攻坚示范区”，12个乡镇实现“减贫摘帽”，减少贫困人口14.08万人。坚持用群众工作统揽信访工作，实行市、县、乡各级领导包案督访消化制度，疑难信访问题总量从2011年的14200件降至400多件。大力开展“三反”和“打黑除恶”专项行动，“平安凉都”创建活动深入推进。安全生产形势总体平稳，事故起数和死亡人数实现“双降”。

　　过去一年，我们切实加强民主法制建设和政府自身建设。自觉接受人大的法律监督、工作监督和政协的民主监督。认真办理人大代表建议和政协提案。高度重视民主党派、工商联和无党派人士的意见和建议，注重发挥工会、共青团、妇联等人民团体的参与监督作用。加强电子政务建设和应用，推进政府信息公开，各类公开办事制度不断完善。“六五”普法深入推进。扎实开展保持党的纯洁性教育试点活动。强化行政监察和审计监督，率先在全省建成公共资源交易中心并投入运行，着力纠正不正之风，勤政廉政建设取得新成绩。

　　各位代表！过去一年取得的成绩实属不易。这是市委、市政府深入研判形势、科学决策、有力应对的结果，是全市各族干部群众凝聚共识、迎难而上、团结拼搏的结果。在此，我代表市人民政府，向为六盘水市经济社会发展作出艰苦努力和付出辛勤劳动的全市广大工人、农民、知识分子、干部，向各民主党派、工商联、无党派爱国人士和各人民团体，向驻市人民解放军、武警官兵、公安干警、民兵和预备役人员，向所有关心和支持我市改革开放和现代化建设的各界人士，表示衷心的感谢并致以崇高的敬意！

　　在充分肯定成绩的同时，我们也清醒地认识到存在的困难和问题。主要是：高速公路建设滞后，乡村道路建设管理差距大，工程性缺水问题突出，生态环境脆弱，制约发展的瓶颈问题没有得到根本解决；产业结构单一，传统产业不强，新兴产业发展缓慢；城镇化率较低，农村公共基础设施落后，城乡居民收入差距大，全面建成小康和扶贫攻坚任务繁重；加强和创新社会管理的任务十分艰巨，人民群众对社会治安状况不够满意，生产安全事故尚未得到有效遏制；政府的履职能力与发展需要和人民要求还有差距，创造力和执行力不够。对于这些问题，我们将采取切实有效措施，下大力认真解决。

　　2013年政府工作的主要任务

　　今年是全面贯彻落实党的十八大精神的第一年，也是“同步小康创建活动”的启动年。做好今年各项工作，保持全市经济社会发展的良好势头，对于如期实现全面建成小康社会的奋斗目标，意义十分重大。

　　今年政府工作的总体要求是：以邓小平理论、“三个代表”重要思想、科学发展观为指导，全面贯彻落实党的十八大、省委十一届二次全会和市委六届二次全会精神，坚持“稳中求快”总基调，围绕“突出特色、做大总量、提速转型、增比进位”总目标，落实“好中求快、快中保好、又快又好、更快更好”总要求，坚定不移地贯彻主基调、实施主战略，坚持走追赶型、调整型、跨越式、可持续发展路子，推进工业化、信息化、城镇化、农业现代化同步发展，以更加开阔的思路、更加解放的思想、更加进取的精神、更加扎实的作风，促进全市经济社会又好又快、更好更快发展，为同步全面建成小康社会奠定坚实基础。

　　全市经济社会发展的主要预期目标是：生产总值增长18%以上；全社会固定资产投资增长50%以上；财政总收入和公共财政预算收入分别增长20%以上；招商引资到位资金增长53%以上；社会消费品零售总额增长20%以上；城镇居民人均可支配收入增长15%以上，农民人均纯收入增长17%以上；人口出生率控制在12.3‰以内；城镇登记失业率控制在4.2%以内；完成省下达的节能减排指标任务。

　　围绕上述总体要求、主要预期目标，我们将按照“坚持三个不能代替”、“突出三个核心指标”、“做到一个大于、一个快于、一个高于”的要求和“515”工作部署，着力做好以下十个方面工作。

　　（一）保持投资较快增长，推动经济加速发展。抢抓国家扩大投资的重要机遇，以国发2号文件为抓手，以“十大工程”为重点，把抓投资作为第一任务，确保完成固定资产投资1600亿元以上，力争完成2000亿元。加强项目前期工作。围绕国家产业政策和投资政策，谋划一批大项目、好项目，调整充实项目库。加强项目研究和规划设计，实行并联审批、联合办公，进一步缩短土地、环评等审查、审批时限。优化项目施工管理。严格实施招投标、项目监理和概预算审核，强化项目跟踪审计。优化施工组织，选择实力雄厚、口碑良好、信誉一流的优强企业，促进项目加快建设。培育和引进一批项目建设专业技术人员。保障项目用地需求。积极争取国家、省增加用地指标，开展城乡建设用地增减挂钩、低丘缓坡土地开发利用，全面清理闲置土地，盘活土地存量，提高项目建设供地率。保证重点建设项目所需环境容量。创新项目融资方式。加大“上争、外引、内聚”的力度，拓宽融资渠道。采取资本金注入、投资补助、贷款贴息等方式，加快直接投资向股权投资、以奖代补、融资平台担保等间接投资转变，撬动金融资本和社会投资。支持市开发投资公司发行二期企业债券。狠抓项目推进实施。强化“五个一”项目推进机制，继续推行“三化”现场观摩和项目集中开工，每季度集中开工300个以上项目，全年新开工1200个以上项目，总投资达到2000亿元以上。加大“旬调度、月督查、季考核”力度，努力形成更多实物工作量。

　　（二）大力推进产业发展，加快结构调整优化。坚持“调特一产、调强二产、调优三产”的思路，打造特色优势产业，努力实现有质量、有效益、可持续的发展。

　　做特做优农业。在稳定粮食总产量的前提下，积极发展喀斯特山区特色农业，提高经济作物和生态畜牧业的比重，拓宽农民增收渠道。发展特色山地农业。全力推进农业十大产业发展，全年新增马铃薯高产示范基地19万亩、商品蔬菜基地6万亩、茶叶15万亩、猕猴桃8万亩、核桃25万亩、油茶5万亩、中药材5万亩、红豆杉3万亩，完成烤烟种植14.5万亩，肉类总产量增长10%以上。推进农业产业化。抓好六枝郎岱现代农业、盘县普古娘娘山生态农业、水城米箩猕猴桃产业、钟山大河都市型现代农业等示范园区建设。培育壮大现有龙头企业，新增一批市级龙头企业。强力推进农业项目招商引资工作，积极开展农业产业招商推介活动。加强农业服务体系建设。加大农业机械、科技培训、质量检测等技术服务力度，夯实现代农业发展基础。实施“千村千社”计划，制定完善支持农民专业合作经济组织的相关配套政策和措施，发展一批农业专业合作社。加快农村信息化平台建设，逐步实现农民与市场的有效衔接。

　　做大做强工业。围绕延长产业链、提高附加值，加快推进我市工业转型升级，实现工业循环发展、可持续发展，全年工业增加值增长24%以上。全力推进工业十大产业发展。着力打造煤电钢、煤电铝、煤电化、煤电材“四个一体化”品牌，确保一批重大项目年内落地。加快煤矿兼并重组和整合技改，加强大型化、基地化、机械化、信息化煤矿和保安电网建设，完成原煤产量8000万吨；支持首钢水钢（集团）公司技改升级，实施双元铝业扩能改造，启动广投盘县铝加工项目；推进六枝电厂、马依电厂等一批火电、风电、水电项目，建成黔桂电厂“上大压小”一台机组、盘北煤矸石电厂以及毛家河水电站、盘县坡上牧场风力发电等电力项目，年内装机容量达835万千瓦，发电量达436亿千瓦时；加快六枝畅达二期、恒远新型建材二期等项目建设，完成水泥产量800万吨、新型墙体标砖6亿块、空心砌块380万立方米；推进老鹰山煤基气化替代燃料二期等煤化工项目；建成西南天地煤炭装备制造、盘江矿山机械制造等装备制造项目；大力培育电子信息、生物制药、节能环保、特色轻工等产业。加快产业园区建设。着力完善园区基础设施、环保设施和标准厂房建设，推动产业向园区聚集。全力抓好六枝路喜循环经济示范、盘县柏果资源精深加工及转化、水城董地煤电铝一体化、钟山水月等一批产业园区建设。新建标准厂房150万平方米以上，完成基础设施投资65亿元、产业项目投资140亿元以上，实现园区工业总产值600亿元以上。把培育负荷、发展高载能产业作为园区发展的切入点，积极推进热电联产项目建设，努力在园区直供电上实现突破，促进能源资源优势转化为经济优势。加强经济运行调节。抓好工业经济监测和预警，建立完善监测调控体系，强化煤电油运等要素保障，确保全年工业经济平稳运行。

　　做新做实服务业。把发展服务业作为产业结构优化升级的重点，结合我市实际，全力推进服务业十大产业发展。大力发展文化旅游产业。围绕承办好第八届全省旅游产业发展大会，发挥“中国凉都”品牌优势，深挖“三线”文化、民族民间文化、消夏避暑文化等资源，着力打造“休闲度假旅游”这张名片。加快凉都体育生态公园、水城野玉海国际山地休闲度假区、六枝牂牁江西嘎景区、盘县妥乐古银杏景区等一批旅游景区景点建设，建成凉都国际、盘江雅阁等一批五星级酒店，规划建设25万平方米小户型休闲旅游度假房。全年接待游客1000万人次，实现旅游综合收入68亿元。加快发展现代物流业。以建设区域性物流中心为目标，推进六盘水煤炭储备中心、钟山区马嘎润发等一批物流项目建设，努力形成专业化、规模化、集约化的现代物流体系。积极拓展其他服务产业。全力改造提升餐饮、住宿等传统服务业。加快培育“楼宇经济”、“会展经济”等新型业态和金融服务、信息服务、研发设计、电子商务等现代服务业。鼓励发展教育、养老、康体、家政等面向民生的服务业。

　　（三）加强城镇体系建设、促进城乡协调发展。坚持做强市中心、做大县城、做特城镇、做美农村“四位一体”，加快城乡一体化发展步伐，全市城镇化率达到45%。注重规划引领。完成第四轮城市总体规划修编工作；加快新一轮控制性详规修编，城市建设用地控制性详规覆盖率达到100%；开展市中心城区各类专项规划编制工作。完成10个小城镇和602个村的村庄整治规划编制工作，实现全市所有乡镇和行政村村庄整治规划全覆盖。加快城镇建设。推进凤凰山、六盘水体育中心、明湖、红桥等一批城市综合体项目建设，抓好老城片区、水西片区旧城和棚户区改造，拓展市中心城区面积5平方公里以上。打造一批特色示范小城镇，每个县（特区、区）建成1至2个特色小城镇，8月底建成3个省级示范小城镇。启动一批具有六盘水特色的工矿区城镇改造。以“美丽乡村”为目标，统筹推进农村危房改造、扶贫生态移民搬迁、“四在农家”建设，全年完成12万户“四在农家”新民居建设和100个村“整村推进”任务。提高承载能力。加快建设市中心城区内环快线、现代有轨电车、大丫口公交始发站、红桥南客运站等重点项目。完善城镇骨干路网、供水、排水、电力、通讯等市政设施和学校、医院、文化等公共设施。完成市中心城区集中供热工程建设150万平方米；基本建成市中心城区和盘县、六枝城区城市排水（雨水）一期工程；启动实施民用燃气保障工程，提升改造市中心城区燃气管网，推进盘县燃气管网建设。强化城市管理。深入开展“五城联创”活动，全面提高城市管理水平。推进城市公交体制改革。加大占道经营、机动车辆乱停乱放等违规行为整治力度。积极推进小街小巷、楼庭院落、车站码头的升级改造和治理。大力实施城市绿化美化亮化工程。加大宣传教育力度，努力提升市民综合素质。建立健全市、县、街道、社区四级城市管理网络和部门联动机制，充分调动社区力量，落实“门前三包”，强化精细化、网格化管理，实现城市管理重心下移。突破城镇化障碍。加快城乡公共服务一体化进程，进一步完善土地流转、社会保障、户籍管理等政策措施，努力消除户籍、住房、教育、社保等方面的门槛，有序推进农业转移人口市民化。积极开展六枝特区撤区设市、盘县撤县设市、水城县撤县设区和乡改镇、镇改办工作。

　　（四）坚持基础设施先行，强力突破发展瓶颈。加快现代综合交通运输体系建设。完成交通投资98.6亿元。配合做好长昆客运专线建设，开工建设六盘水至安顺城际铁路；建成水盘高速、六镇高速，加快杭瑞高速境内段、六六高速、机场高速建设，开工建设盘兴高速、六威高速；完成212省道窑上至俄脚段改造，建成纸厂至红桥、长寨至兴隆、英武至大山二级公路，开工建设郎岱至毛口旅游公路；开工建设通村油路1111公里，建成1261公里；建成六盘水月照机场，启动盘县机场前期工作；建成野钟新发、西嘎旅游码头，开工建设毛口、岩脚等旅游码头。加快解决工程性缺水问题。完成水利投资50亿元以上。配合抓好黔中水利枢纽一期工程建设，加快双桥、旧院、卡河、朱昌河、懒龙河、观音岩、出水洞等中型水库建设，开工建设纳革、彭家桥、毛草寨、五里坪等一批小型水库，全面开展纳入国家“三位一体”规划水利项目前期工作并力争早日开工。完成烟水配套工程2万亩，治理水土流失面积48平方公里，完成中小河流域治理50平方公里，治理病险水库3座，新增解决22万农村人口饮水安全问题。全力支持信息基础设施和电网建设。抓住国家将我市列为第一批“智慧城市”试点机遇，加强信息保障能力建设，完成信息基础设施投资5.8亿元。新增50个自然村寨通电话和宽带，建成覆盖39785户农村广播电视“村村通”和200516户农村广播电视“户户通”直播卫星接收站，完成825个行政村有线广播电视联网延伸覆盖。推进城乡电网建设和改造，完成投资7.27亿元。

　　（五）扩大开放深化改革，推进体制机制创新。把扩大开放放在更加重要的位置，扎实推进“改革开放年”活动。扩大开放方面，努力构筑对外开放新高地。加快六盘水海关、检验检疫机构、口岸物流中心、“无水港”等开放载体建设；加强与对口帮扶城市、国内外友好城市的交流与合作，积极构建“攀西—六盘水”经济区、毕水兴能源资源富集区。着力打造对外开放新平台。积极参加第九届泛珠三角区域合作与发展暨经贸洽谈会、省酒博会、川滇黔十二市州合作与发展峰会等活动，高标准承办好“第八届贵州省旅游产业发展大会”暨“第十届中国凉都·六盘水消夏文化节”，以凉都品牌助推旅游；精心筹备各类大型商务会展、文体活动和高端论坛等活动，提高办节办会水平。大力培育对外开放新主体。打破所有制、行业和地域界限，推进企业整合重组、强强联合，加快建设一批大企业、大集团；继续实施民营经济“三年倍增计划”，鼓励民营经济进入基础产业、金融服务、文化产业和商贸流通领域，重点扶持发展一批具有良好前景的中小民营企业，努力实现民营经济增加值占生产总值比重提高到48%以上。全力增创招商引资新优势。完善招商引资激励措施，大力推行产业招商、园区招商、以商招商，加强与世界500强、大型民企和央企的合资合作，引导更多战略投资者落户我市；加强招商引资项目跟踪和协调服务，提高招商项目“四率”，确保全年引进市外实际到位资金1000亿元以上。

　　深化改革方面，推进国有企业改革，制定出台国有企业改革改制方案，推动市、县两级国有企业改革脱困、加快发展。加大“引金入市”力度，积极引进股份制银行、外资银行、证券公司、基金公司等各类金融机构，支持市内企业通过上市、发行债券等方式到资本市场直接融资。推进农村综合改革，开展农村土地所有权、土地承包经营权、房屋所有权确权颁证试点工作。实质推进国库集中支付制度。深化医药卫生、教科文等社会事业领域体制改革及事业单位分类改革。认真落实支持县域经济发展和扩权强县的各项政策措施，支持有条件的县区冲刺全国百强县和贵州省经济强县，推动县域经济壮大规模、增强实力。

　　（六）加强生态环境保护，切实保障持续发展。把生态文明理念融入经济建设、政治建设、文化建设、社会建设各方面和全过程，加快建设“美丽六盘水”，努力实现天蓝、水净、山绿。加快国家循环经济示范城市建设。立足我市的产业特点和资源现状，按照减量化、再利用、资源化的原则，积极构建企业、园区、社会三个层面的循环经济体系，出台市级鼓励发展循环经济的政策措施，重点抓好煤矸石、粉煤灰、煤层气、冶金渣、焦炉煤气、矿井废水及城市“中水”等综合利用，努力走出一条具有六盘水特色的循环经济发展路子。大力实施生态修复和国土绿化行动。完成营造林35万亩以上，治理石漠化17.6平方公里以上，森林覆盖率提高2个百分点以上。加强环境综合治理，严格保护和管理自然保护区、重点水源区、生态脆弱区，坚决禁止乱挖滥采矿产资源，加强重点流域污染防治，强化工矿区环境综合整治，加快乡镇污水处理厂建设，大力整治村庄村寨环境。扎实抓好节能减排。严格执行环保、安全、能耗等市场准入标准，建立健全淘汰落后产能工作目标责任评价、考核和奖惩制度，落实政府和企业责任，运用经济、法律、行政等手段，全年淘汰落后产能204万吨、节能80万吨标煤以上；确保燃煤电厂、水泥企业脱硝设施建成投运，水钢（集团）公司脱硫设施完成调试并稳定运行。加强生态文明制度建设。加大《清洁生产促进法》宣传贯彻力度，促进生产、生活、流通等各环节的文明清洁生产。开展绿色园区、绿色工厂、绿色城市、绿色社区、绿色家庭创建活动，倡导低碳环保的生产生活方式。认真落实生态补偿机制，开展地质灾害综合防治和矿山环境恢复治理，加快建立反映市场供求和资源稀缺程度、体现生态价值和代际补偿的资源有偿使用制度和生态补偿制度。

　　（七）大力发展社会事业，完善公共服务体系。优先发展教育事业。坚持教育投入优先，全市行政经费压缩5%，用于支持“9+3”义务教育和三年免费中等职业教育计划的实施，确保小学辍学率低于2%、初中辍学率低于3%。继续推进“四项突破工程”。加大农村中小学学生营养餐工程实施力度。开工建设农村寄宿制学校学生宿舍4万平方米，闲置校舍改建幼儿园9所，农村小学、教学点增设附属幼儿班45个，新建、改建进城务工人员子女义务教育学校4所，新建、扩建高中、中职学校各4所，开工建设六盘水师范学院二期、六盘水职业技术学院二期工程。依托大企业职教优势，建设一批大中专层次的职业院校。加大教师培训力度，强化教师、学生思想道德建设，全面推进素质教育。大力扶持民办教育、民族教育、特殊教育、边远山区教育，均衡发展各类教育。加快发展医疗卫生事业。巩固提高新农合参合率，全面推广商业保险机构参与新农合经办服务及大病补充保险工作。开工建设市人民医院二期工程和市中医院，建好县级人民医院，完成19所乡镇卫生院标准化建设。大力培养以全科医生为重点的基层医护人员，启动实施“千名乡村医护人员培训”、“万名医师支援农村”工程；抓住浙江医疗卫生系统与我省结对帮扶的机遇，大力培训和引进优秀人才，提升医疗服务水平。大力发展科技文化体育事业。加快建设以企业为技术创新主体的科技创新体系，积极实施“686”科技创新工程，提高科技进步水平；以科技带动产业发展，规划建设一批科研站所，推进贵州科学院六盘水分院建设。完善城乡公共文化服务体系，加快市级“七馆四中心”、县级“四馆两中心”建设，大力实施广播电视“村村通、户户通”等广电惠民工程，启动六盘水体育中心二期工程、市文化馆、老王山多梯度高原运动训练基地建设，基本建成六盘水大剧院、六盘水体育中心、市图书馆、市博物馆、六盘水美术馆。举办六盘水市体育运动会。加强文化遗产和自然遗产保护。统筹推进人口计生等工作。进一步夯实人口计生基层基础，巩固扩大计生工作成果，确保“三降一升”目标实现，力争再进位。创新人才引进机制，完善配套措施，强化人才队伍培养，营造良好用人环境。切实做好妇女儿童、民族、宗教、外事、侨务、老龄、对台、档案、地方志、红十字等工作。

　　（八）注重保障改善民生，提高人民生活水平。紧扣“全面建成小康社会”主题，全力办好“二十件民生实事”。扎实推进扶贫开发。围绕集中连片特殊困难地区区域发展和扶贫攻坚规划，实施扶贫生态移民搬迁2万人，减少贫困人口15.2万人，新增19个贫困乡“减贫摘帽”，完成52个贫困村“整村推进”计划。继续加强“减贫摘帽”贫困乡脱贫致富工作。积极扩大社会就业。坚持以产业促就业、以创业带就业，千方百计实现城乡居民收入倍增。做好申报国家级创业型城市创建工作。全面落实创业扶持政策，完成扶持微型企业3000户。统筹做好高校毕业生、农村转移劳动力、就业困难人员、退役军人的就业和技能培训工作。全年新增城镇就业10万人。完善社会保障体系。进一步扩大城镇职工基本医疗、基本养老、失业、工伤、生育保险参保人数，继续巩固和扩大新型农村和城镇居民社会养老保险参保人数。完善社会救助和保障标准与物价上涨挂钩联动机制，做好城乡居民最低生活保障提标工作，确保保障标准提高10%以上。实施23.8万受灾困难群众冬春口粮救助。改进困难群众生活用煤供应方式。加强保障性安居工程建设。新建保障性安居工程35700套，其中廉租房1096套、公共租赁住房9262套、城市棚户区15000套、煤矿棚户区10342套；竣工保障性安居工程13974套，完成农村危房改造28080户；继续做好公积金贷款支持保障性安居工程试点工作。

　　（九）加强社会管理创新，维护社会和谐稳定。提升社会管理服务能力和水平。学习借鉴外地先进经验，大力开展“和谐社会模范区”创建活动，建立健全重大决策社会稳定风险评估机制，推广社区戒毒、社区康复“阳光工程”，倡导“一线工作法”，推动全市社会管理向全省一流水平迈进。继续完善领导干部公开接访、包案督访、带案下访制度，加快解决一批与群众利益密切相关的突出问题。注重关爱弱势群体。开展关爱外出民工、留守儿童、空巢老人“三关爱”活动，加强对流浪儿童和乞讨人员的救助管理，重视“失独家庭”养老扶助，健全残疾人社会保障服务体系。加强公共安全管理。牢固树立安全发展理念，强化政府的监管责任和企业的主体责任，扎实推进安全标准化建设和安全生产信息化建设，持续深入开展“打非治违”专项行动，重点抓好煤矿、道路交通、消防安全、食品药品等领域和行业专项整治，坚决遏制重特大事故的发生。扎实推进“平安六盘水”建设，建成“天网工程”，大力开展“打黑除恶”、打击“两抢一盗”专项行动，加大社会面巡逻防控力度，努力提高人民群众安全感和满意度。加强国家安全、司法行政、双拥优抚等工作。

　　（十）加强政府自身建设，提高政府工作水平。完成今年各项目标任务，需要全市政府系统付出艰辛努力。我们将按照“团结、务实、勤奋、清廉”的要求，严格执行中央、省委和市委改进工作作风、密切联系群众的规定，加强服务政府、效能政府、法治政府、责任政府建设。进一步解放思想激发创造力。围绕“十破十立”和“十强化十转变”，坚决摆脱“旧思维”、突破“老框框”、改变“潜规则”，敢于尝试、敢于担当、敢想会干，努力实现增比、进位、突破，开创改革发展新局面。进一步转变职能提升服务力。强化“环境是对外开放第一竞争力”的理念，深化行政管理体制和审批制度改革，能减则减、能放则放、能快则快、能优则优，为群众和企业提供便捷高效的政务服务。加强效能建设，治庸治懒治散，严肃查处不作为、慢作为、乱作为和“吃、拿、卡、要”等违法违规行为，做到在其位、谋其政、尽其责。进一步抓好落实强化执行力。令行禁止，雷厉风行，确保中央、省委和市委的决策部署落实到位。主动作为，不等不靠，积极争取政策，用足用好政策。深入基层，深入群众，及时采取有效措施排忧解难。讲求实干，注重实效，坚决反对形式主义、官僚主义。大力精简会议、文件和应酬，腾出更多的精力抓落实。进一步依法行政彰显公信力。自觉接受市人大的法律监督和工作监督、市政协的民主监督以及新闻舆论和社会监督。高度重视民主党派、工商联和无党派人士的意见和建议，充分发挥工、青、妇等人民团体的桥梁纽带作用。严格行政执法，强化责任追究，推进政务公开、政府信息公开。加强行政监察和审计监督，注重反腐倡廉教育，自觉遵守廉政准则，秉公用权、依法用权、廉洁用权。大力提倡艰苦奋斗、勤俭节约，反对挥霍奢侈、铺张浪费，以廉洁的政府形象取信于民。

　　各位代表！完成今年的目标任务，实现对人民群众的庄严承诺，使命崇高，责任重大。让我们紧密地团结在以习近平同志为总书记的党中央周围，全面贯彻落实党的十八大精神，在市委的坚强领导下，进一步解放思想，开拓进取，凝心聚力，真抓实干，向着全面建成小康社会宏伟目标阔步前进，为谱写六盘水人民美好生活的新篇章而努力奋斗！
